# High Throughput Automated Allele Frequency Estimation by Pyrosequencing

**DOI:** 10.1371/journal.pone.0002693

**Published:** 2008-07-16

**Authors:** Julie Doostzadeh, Shadi Shokralla, Farnaz Absalan, Roxana Jalili, Sharareh Mohandessi, James W. Langston, Ronald W. Davis, Mostafa Ronaghi, Baback Gharizadeh

**Affiliations:** 1 The Parkinson's Institute, Sunnyvale, California, United States of America; 2 Stanford Genome Technology Center, Stanford University, Palo Alto, California, United States of America; 3 Department of Molecular Biology, Princeton University, Princeton, New Jersey, United States of America; University of Chicago, United States of America

## Abstract

Pyrosequencing is a DNA sequencing method based on the principle of sequencing-by-synthesis and pyrophosphate detection through a series of enzymatic reactions. This bioluminometric, real-time DNA sequencing technique offers unique applications that are cost-effective and user-friendly. In this study, we have combined a number of methods to develop an accurate, robust and cost efficient method to determine allele frequencies in large populations for association studies. The assay offers the advantage of minimal systemic sampling errors, uses a general biotin amplification approach, and replaces dTTP for dATP-apha-thio to avoid non-uniform higher peaks in order to increase accuracy. We demonstrate that this newly developed assay is a robust, cost-effective, accurate and reproducible approach for large-scale genotyping of DNA pools. We also discuss potential improvements of the software for more accurate allele frequency analysis.

## Introduction

Population-based studies are commonly used to locate genes that underlie complex diseases in genetic association studies, which have shown to be a crucial tool for mapping complex diseases and traits. Although the cost of individual SNP genotyping has been reduced significantly, the use of DNA pooling decreases the cost even further, especially for large-scale genetic studies.

Pyrosequencing [1] is a DNA sequencing method, which allows quantitative measurement of population allelic frequencies [2]–[6]. Although Pyrosequencing has shown to be a robust and relatively accurate method for such studies, the pipetting consistency is a crucial question due to random and manual errors, which affects the accuracy. Moreover, the cost of primer biotinylation per amplification is relatively high particularly for large-scale studies. Another issue that also affects the accuracy of quantitative genotyping is the intensity of sequence signal peaks generated during the incorporation of nucleotide dATP-alpha-thio that are generally 10–15% higher.

In this study, we have addressed these issues by developing a robust, cost-effective, accurate and reproducible assay for large-scale genotyping of DNA pools based on a combination of robotic DNA pooling, universal biotin amplification, touchdown PCR, using lower DNA concentrations, and finally replacing dATP-alpha-thio wtih dTTP readouts by redesigning the genotyping for accurate peak uniformity. The assay is remarkably cost-effective and has a general approach.

## Methods

### Patients and DNA extraction

192 patients with Parkinson's disease (PD) and 192 control individuals were enrolled in this study. A consent form was signed by all patients participating in this project. Genomic DNA from PD patients and control individuals were extracted from blood and quantified by spectrophotometer (Qiagen, Hilden, Germany). DNA quality was verified by both Gel electrophoresis and spectrophotometer in order to evaluate DNA integrity and any possible contamination of DNA samples by RNA or protein. To confirm the quality of DNA, no degradation by electrophoresis gel and a 260/280 ratio between 1.7 to 2 for all extracted DNA were required [7].

### DNA pooling

The initial concentration of each DNA sample from both controls and cases was 15 ng/μl. The DNA concentration measurements were performed by NanoDrop ND spectrophotometer (NanoDrop Technologies, Wilmington, DE). All the samples were robotically diluted to 1.5 ng/μl with TE buffer (10 mM Tris, bring to pH 8.0 with HCl and 1 mM EDTA) using Biomek FX Dual Bridge Laboratory Automation Workstation (Beckman Coulter, Fullerton, CA). From the diluted samples, 10 μl of 192 controls were robotically pooled and combined from 96-well Thermo-Fast microplates (Abgene, Surrey, Uk) into Deep Well titer plates (Beckman, Fullerton, CA). The same procedure was applied separately to the 192 DNA samples from the cases to have a final pooled DNA concentration of 1.5 ng/μl (15 ng/10μl). For evaluation of the dilutions, control and case samples were also separately pooled to 96, and 192 final dilutions robotically.

### Universal biotin amplification

The amplification primers were designed by the online software SOP^3^ version 2 (http://imgen.ccbb.pitt.edu/sop3/). A universal 22-mer (5′-CCG AAT AGG AAC GTT GAG CCG T) adopted from [8] was added to the 5′ end of the primer designated for biotin-labeling [9]. A 22-mer universal biotin primer (UBP) with the same sequence was synthesized by 5′-end biotin labeling for universal biotinylation. All the primers were synthesized in-house. PCR was performed in 50 μl reactions by GenAmp 9700 (Applied Biosystems, Foster city, CA). Each 50 μl PCR reaction contained 15 ng pooled DNA, 25 μl pre-prepared PCR mix HotStarTaq Master Mix kit (Qiagen, Hilden, Germany), 10 pmole forward, 1 pmole reverse pimer, 9 pmole of universal biotin primer, and water. The touchdown PCR conditions was initiated with heated lid 95°C 15 min, followed by 15 cycles of 94°C 1 min, and 60°C 1 min, 72°C 1min, and then 30 cycles of 94°C 1 min, 53°C 1 min, 72°C 1min and a final 72°C 10 min and 4°C hold. For samples that were not amplified by this protocol the touchdown annealing temperatures were 62°C and 50°C.

### Pyrosequencing

Sequencing primers were designed by SOP^3^. Single strand preparation and sequencing primer hybridization were performed semi-automatically using a Vacuum Prep Tool and Vacuum Prep Worktable (Biotage AB, Uppsala, Sweden) as described before [10]. Pyrosequencing was performed on an automated plate-based bench-top PSQ™HS96A system at a dispensing pressure of 625 mbar with 4 ms open time and 65 sec cycle time. The nucleotide dispensation order was set for each SNP. The sequencing primers and the pyrosequencing SNP dispensation orders can be found online table A on the following website http://www-sequence.stanford.edu:16080/pyrosequencing. The sequence results were obtained in pyrogram formats.

## Results and Discussion

In order to achieve high precision pooling with minimal sampling errors and same systemic error, Biomek automation workstation was used to separately pool 192 controls and 192 cases with a final concentration of 15 ng/μl. For assay accuracy evaluation, pools of 96 and 192 control samples were also prepared.

For universal biotin amplification a 22-mer sequence that has no interaction with human genome was selected from a previous study [8] and tagged to one of the two amplification primers designated for biotinylation. Another primer with the 22-mer tag was biotin-labeled for general biotin amplification (a total of three primers were used in each PCR reaction). Amplification primers were designed for 230 SNPs using the same universal 22-mer tag for all. The respective pools of 192 controls and 192 cases were amplified separately for the 230 SNPs. 203 out of 230 SNPs yielded PCR products (figure S1). By decreasing the temperature from 53°C to 50°C in touchdown PCR, we were able to amplify the samples that were challenging in the amplification. These SNPs have been listed online with * in table A online

**Figure 1:**
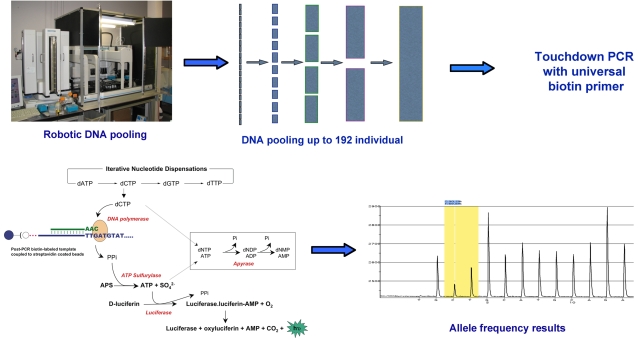
The overall workflow for high throughput allele frequency determination.

The amplicons were prepared for DNA sequencing (single-strand separation and sequencing primer annealing) by Vacuum Prep WorkStation using 10 μl of each PCR product. The primed amplicons were sequenced and genotyped by the high sensitive pyrosequencer requiring lower amounts of sample and reagents. The genotyping results were analyzed by Software HS96A version 1.2. The genotyping results of the test pool samples were also analyzed manually to investigate the accuracy of the software. Figure 1 demonstrates the overall procedure.

**Figure 2:**
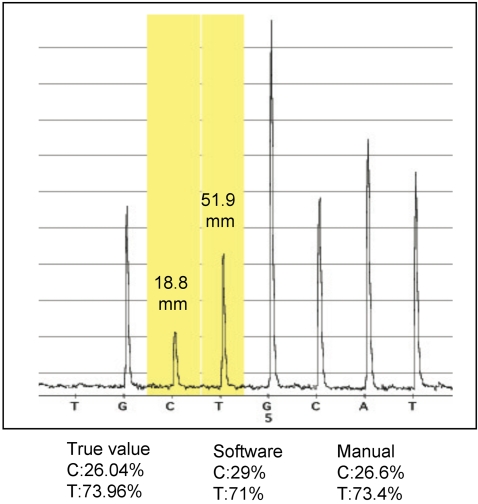
Shows the accuracy comparison of manual and software analysis with reference to true SNP values of the COMT gene. Each pool consists of DNA from 192 individuals. The pyrograms signal peak heights analyzed with reference demonstrates a) low error rate b) higher error rate between manual and software analysis.

To evaluate the accuracy of the assay, for 3 SNPs (CYP2E1[rs:915906], DrD2[rs:6279] and COMT[rs:933271]), pyrosequencing was performed on 192 control samples individually to obtain the true SNP genotypes. For these SNPs pools of 96 and 192 were genotyped by pyrosequencing as described above. The obtained results were compared with the individual genotyping results. Table 1 shows the pooling error rates for the three SNPs in 96-control pools 0.55%, 0.9% and 1.55% and the 192-control pool 0.78%, 1.68% and 0.55% respectively. The evaluation was performed manually by using a ruler precisely measuring the peak heights. All the samples were sequenced in triplicate. We found out the allele frequency analysis software was not as accurate as the manual evaluation. As indicated in Table 1, the allele frequency error rates are significantly higher using the software evaluation. The mean and standard deviation for software analysis are 2.24 and 1.40 (median 2.1) where the respective mean and standard deviation for manual analysis are 1 and 0.5 (median 0.84). Figure 2 shows the allele frequency difference error rates between manual and software analysis for a SNP in COMT[rs:933271] gene in a pool of 192 controls. We speculate that the software makes adjustments for peak height measurements based on signal intensity drops. The statistical analysis suggests that the software needs to be improved for more precise analysis. We recommend manual evaluation for higher precision until the software is improved.

**Table 1:** Comparison of allele frequency of three SNPs from a pool of 192 controls using manual and software analysis

Gene name: CYP2E1, SNP ID: rs 915906								
No.of Samples	Individual Samples		Pooled Samples (Software)		Pooled Samples (Manual)		% Error (Software)	% Error (Manual)
	C%	T%	C%	T%	C%	T%		
1-96	18.75	81.25	18.8	81.2	18.2	81.8	0.05(±4.42)	0.55(±0.063)
1-192	16.4	83.6	14.2	85.8	15.62	84.38	2.2(±4.72)	0.78(±0.042)
Gene name: DrD2, SNP ID: rs 6279								
No.of Samples	Individual Samples		Pooled Samples (Software)		Pooled Samples (Manual)		% of Error (Software)	% of Error (Manual)
	C%	G%	C%	G%	C%	G%		
1-96	28.64	71.35	30.5	69.5	29.55	70.45	1.86(±0.49)	0.9(±0.065)
1-192	31.9	68.1	36.1	63.9	33.58	66.42	4.2(±1.42)	1.68(±0.034)
Gene name: COMT, SNP ID: rs933271								
No.of Samples	Individual Samples		Pooled Samples (Software)		Pooled Samples (Manual)		% of Error (Software)	% of Error (Manual)
	C%	T%	C%	T%	C%	T%		
1-96	26.04	73.96	27.98	72.02	27.59	72.41	1.94(±6.59)	1.55(±0.18)
1-192	26.04	73.96	29.25	70.75	26.59	73.4	3.21(±4.40)	0.55(±0.12)

Manual and software analysis comparison with the reference (true values) for pools of 96 and 192 for three SNP from genes CYP2E1, DrD2 and COMT. The table demonstrates significant lower error rates by manual evaluation.

The 192 pool of cases and 192 pool of controls were genotyped for 230 SNPs and the results of these genotyping results are available on the above-mentioned website. Amplification primer sequences and sequencing primers are available on Table A, and the dispensation orders are also listed as Table B online. The name all of SNPs, their positions, genotyping results for control and cases and the control-case difference are listed on the online Table C.

In our study we avoided using nucleotide A sequence signal peaks. In Pyrosequencing, the intensity of nucleotide dATP-alpha-thio signal peak is usually 10 to 15 percent higher than other nucleotides [11], which results in inconsistent non-uniform signal peaks for allele frequency analysis. We have approached this problem by using the complementary strand in all our SNP allele frequency experiments (hence, the complementary strand should be biotin-labeled).

For manual analysis of SNPs in the repeat regions, there is sometimes lack of an adjacent single base before the SNP for accurate measurement. To address this, we recommend measuring the next single base peak height in the pyrogram after the SNP for correct allele frequency determination.

In conclusion, we have developed an automated high throughput assay for large-scale DNA pool analysis for allele frequency estimation and determination. The assay is highly robust, accurate and cost-effective. The universal biotin amplification has a general approach and could be used for studies of any scale. The assay addresses the challenges that can increase the accuracy and precision of allele frequency estimation. Although not all the labs might have access to the robotic sample pooling, this could most likely be outsourced. The cost efficiency for biotin-labeling and DNA pooling decreases the cost by many orders of magnitude, which allows many large scale studies possible. Furthermore, the pooled DNA samples could be stored for future analysis of other relevant markers.

## Supporting Information

Figure S1 Gel staining figure of different SNPs amplified with universal biotin sequence tag from genomic DNA.
(10.29 MB TIF)
