# Supplementary figures and images for: High Throughput Automated Allele Frequency Estimation by Pyrosequencing
Source: PLoS One. 2008 Jul 16;3(7):e2693. doi: 10.1371/journal.pone.0002693 (PMC2442187; doi:10.1371/journal.pone.0002693)

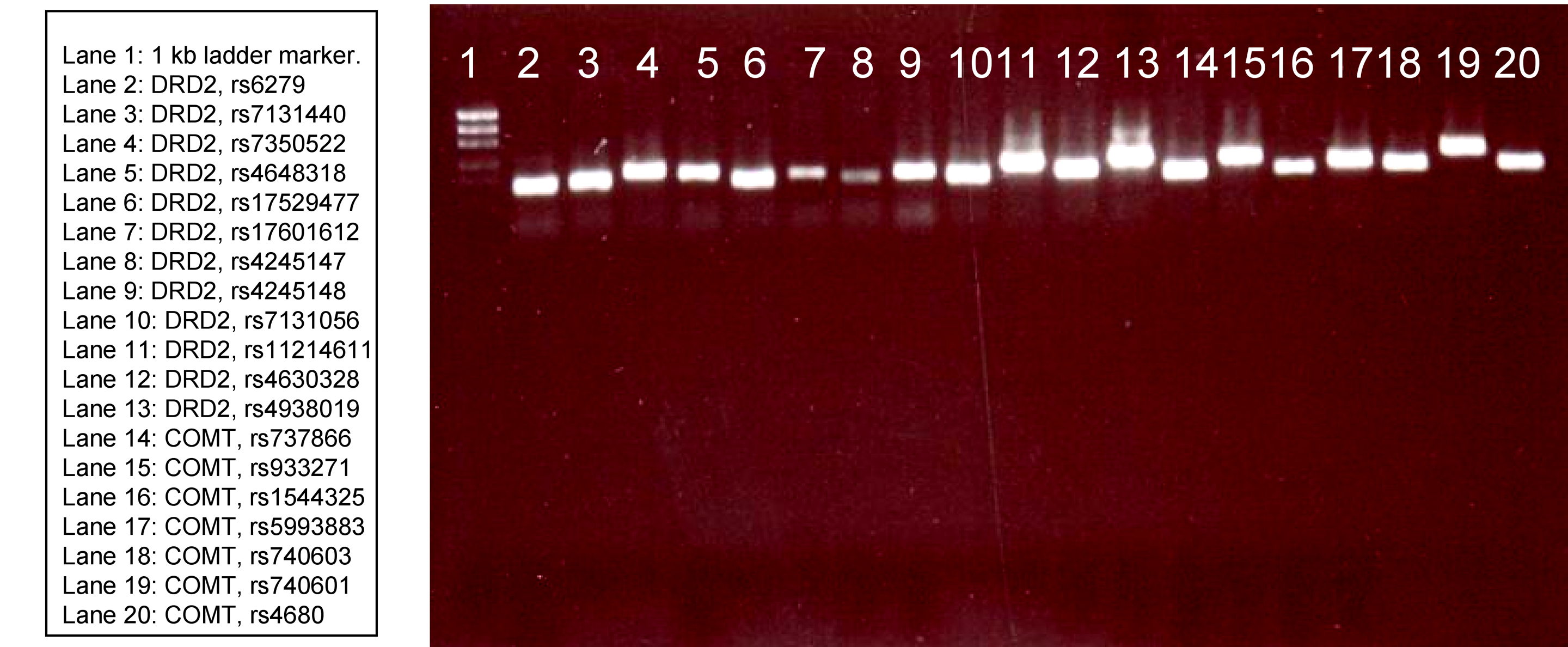

Supplement: Figure S1 — Gel staining figure of different SNPs amplified with universal biotin sequence tag from genomic DNA. (10.29 MB TIF) [file pone.0002693.s001.tif]
